# Lipoprotein Genotype and Conserved Pathway for Exceptional Longevity in Humans

**DOI:** 10.1371/journal.pbio.0040113

**Published:** 2006-04-04

**Authors:** Gil Atzmon, Marielisa Rincon, Clyde B Schechter, Alan R Shuldiner, Richard B Lipton, Aviv Bergman, Nir Barzilai

**Affiliations:** **1**Institute for Aging Research, Albert Einstein College of Medicine, Bronx, New York, United States of America; **2**Diabetes Research and Training Center, Albert Einstein College of Medicine, Bronx, New York, United States of America; **3**Department of Pediatrics, Albert Einstein College of Medicine, Bronx, New York, United States of America; **4**Department of Family and Social Medicine, Albert Einstein College of Medicine, Bronx, New York, United States of America; **5**Department of Neurology, Albert Einstein College of Medicine, Bronx, New York, United States of America; **6**Department of Epidemiology and Population Health, Albert Einstein College of Medicine, Bronx, New York, United States of America; **7**Department of Pathology, Albert Einstein College of Medicine, Bronx, New York, United States of America; **8**University of Maryland School of Medicine, and the Geriatrics Research and Education Clinical Center, Baltimore Veterans Administration Medical Center, Baltimore, Maryland, United States of America; Buck InstituteUnited States of America

## Abstract

Alteration of single genes involved in nutrient and lipoprotein metabolism increases longevity in several animal models. Because exceptional longevity in humans is familial, it is likely that polymorphisms in genes favorably influence certain phenotypes and increase the likelihood of exceptional longevity. A group of Ashkenazi Jewish centenarians ( *n* = 213), their offspring ( *n* = 216), and an age-matched Ashkenazi control group ( *n* = 258) were genotyped for 66 polymorphisms in 36 candidate genes related to cardiovascular disease (CVD). These genes were tested for association with serum lipoprotein levels and particle sizes, apolipoprotein A1, B, and C-3 levels and with outcomes of hypertension, insulin resistance, and mortality. The prevalence of homozygosity for the −641C allele in the *APOC3* promoter (rs2542052) was higher in centenarians (25%) and their offspring (20%) than in controls (10%) ( *p* = 0.0001 and *p* = 0.001, respectively). This genotype was associated with significantly lower serum levels of APOC3 and a favorable pattern of lipoprotein levels and sizes. We found a lower prevalence of hypertension and greater insulin sensitivity in the −641C homozygotes, suggesting a protective effect against CVD and the metabolic syndrome. Finally, in a prospectively studied cohort, a significant survival advantage was demonstrated in those with the favorable −641C homozygote ( *p* < 0.0001). Homozygosity for the *APOC3* −641C allele is associated with a favorable lipoprotein profile, cardiovascular health, insulin sensitivity, and longevity. Because modulation of lipoproteins is also seen in genetically altered longevity models, it may be a common pathway influencing lifespan from nematodes to humans.

## Abstract 2

An allele of apolipoprotein C3 (which regulates lipoprotein metabolism) is more prevalent in centenarian humans than controls. This genotype is associated with a favorable lipoprotein profile, cardiovascular health, insulin sensitivity, and longevity.

## Introduction

Manipulation of single genes can confer longevity in lower organisms, supporting the notion that understanding pathways for exceptional longevity may be possible in humans. Because the contribution of genetics to lifespan is estimated to be only ˜20%, a way to enhance the odds of identifying genetic factors is to study those with very exceptional survival [ 1]. Favorable modulation of aging processes and disease susceptibility is strongly inherited in families with exceptional longevity [ 2, 3]. Current centenarians have survived to more than double their life expectancy at birth, and have often been spared from age-related diseases, specifically cardiovascular disease (CVD), Alzheimer disease, diabetes mellitus (DM), and cancer [ 4]. Furthermore, evidence suggests that the offspring of centenarians may inherit protection against major causes of mortality, because they have approximately 50% lower prevalence of these major diseases than control groups [ 5].

Because of the strong heritability of this survival advantage, we began a search for gene variants that could explain the extended life of centenarians and their families among a genetically homogeneous population of Ashkenazi Jews. Crucial to our approach is the knowledge that only 1/10,000 survives to 100 y, while the average lifespan in the US is ˜77 y. Thus, enrichment in the frequency of certain gene variants at extreme old age probably reflects a selection effect that increases the likelihood of survival. Here we test if polymorphisms in certain candidate genes are significantly more frequent in centenarians than in younger participants. Given that the extreme old age of the probands precludes the use of a proper age-matched control group for analysis of traits that may be related to exceptional longevity, (e.g., lipoprotein levels), we also recruited their offspring, many of whom presumably inherited longevity gene variants and thus should also manifest the longevity phenotype. We reasoned that this approach would permit comparison of the offspring to age-matched controls who would not be expected to be enriched with longevity gene variants, thereby accounting for period and cohort effects and phenotypic variation that may occur at older age [ 6].

We recently reported that lipoprotein particle sizes are increased in centenarians and their offspring, and suggested that this trait may increase the likelihood of attaining exceptional longevity [ 6]. This phenotype was also associated with approximately a 3-fold increased frequency of homozygosity (valine–valine [VV]) of a common functional variant (I405V) in the cholesterol ester transfer protein *(CETP)* gene, and decreased CETP levels. Further, through a genome-wide scan, Geesaman and coworkers found increased frequency of polymorphisms in the microsomal transfer protein in centenarian sibling pairs but not in other populations [ 7]. Here we implicate a polymorphism in the apolipoprotein C3 gene *(APOC3),* yet another gene regulating lipoprotein metabolism, in human longevity. We further report association of this same polymorphism with decreased serum apolipoprotein C3 (APOC3) levels, and favorable lipoprotein profile, outcomes of age-related disease, and lifespan.

## Results

**Figure 1:**
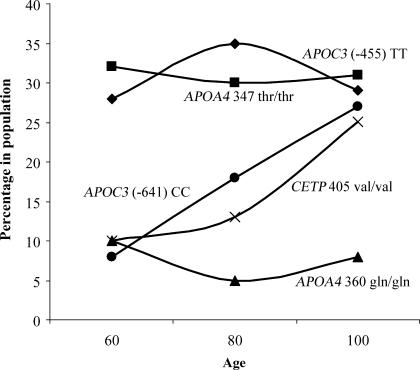
Distribution of “Favorable” Gene Polymorphisms of *APOC3, APOA4,* and *CETP* in the Study Population The genotype frequencies of the favorable gene polymorphisms of *APOC3 C(*− *641)A* and *CETP* I405V were analyzed in controls and probands (60 to 100 y old). (Offspring were not included in this analysis due to the genotype dependency of this group on proband genotypes.) The frequency of these two variants was found to be higher among centenarians, with a monotonic increase with age. Other polymorphisms in these same genes ( *APOC3* (−455) TT, *APOA4* 347 TT, and *APOA4* 360 EE) showed no differences in genotype frequency with age.

**Figure 2:**
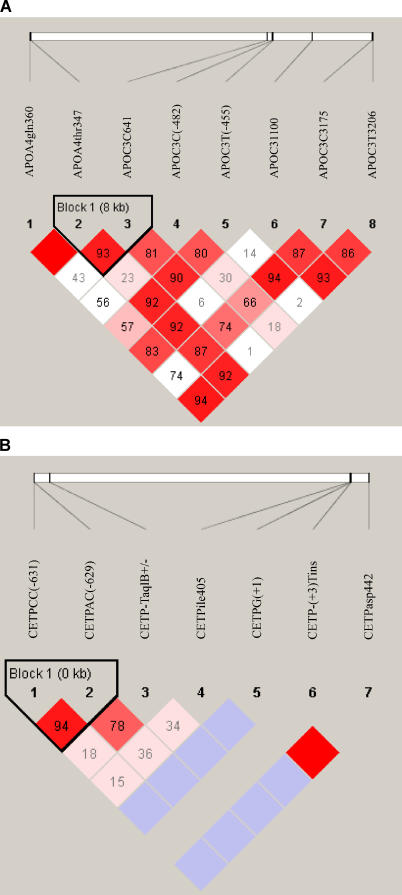
Haplotype Structure of *APOC3* and *APOA4* and *CETP* Ordinal arrangement of 15 SNP associated with CVD and lipoprotein metabolism, according to their position on chromosomes with LD (number in boxes) where the highest rate is represented in red and no LD in lilac. Blocks define potential haplotypes between two clustered genes. (A) *APOC3* and *APOA4.* (B) *CETP.*

**Figure 3:**
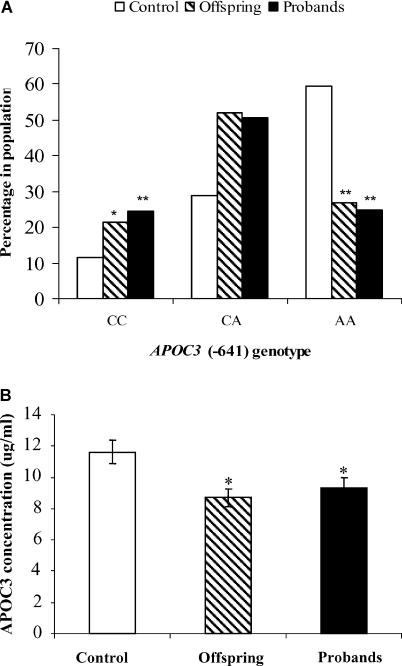
Genotype Distribution of *APOC3* C(−641)A and Serum APOC3 Concentrations (A) *APOC3* C(−641)A. (B) Serum APOC3. * *p* < 0.05, ** *p* = 0.001 versus control. CC, homozygous for (−641) C; AA, homozygous for (−641) A; CA, heterozygous for (−641) C/A.

We reasoned that due to selective mortality, favorable genotypes will be present at higher frequency among centenarians than in younger controls. We studied 66 single nucleotide polymorphisms (SNP) in 36 candidate genes in pathways related to lipoprotein metabolism and other risk factors for CVD. After applying the Bonferroni correction for multiple comparisons, two of the associations with exceptional longevity remained statistically significant. Analysis of the frequency trend with age (in centenarian and control groups combined) for polymorphisms in *APOC3* and *CETP* ( Figure 1), revealed a highly significant ( *p* < 0.0006; β = 1.89 [95% CI, 0.2–3.6]) monotonically increasing frequency trend for homozygosity for the *APOC3* −641 C allele (CC)(rs2542052), and near-significance (β = 3.06 [95% CI, 0.33–5.8, *p* = 0.069]) for homozygosity for the *CETP* codon 405 valine allele (VV)(rs5882). The CC genotype at position −641 of *APOC3* had a prevalence of 25% among the centenarians and 20% in their offspring compared with only 10% in controls ( *p* = 0.0001 and *p* = 0.001, respectively). Five additional SNPs in *APOC3* and six SNPs in *CETP* ( Figure 2) showed no evidence of association with longevity. Conversely, the prevalence of the *APOC3* AA genotype was reduced in both centenarians and their offspring compared with controls ( *p* = 0.001 both) ( Figure 3A).

Linkage disequilibrium (LD) analysis of the *CETP* gene revealed that the single nucleotide polymorphism rs1800776 was in LD with rs1800775 just 2 bp apart (D′ = 0.94; r ^2^ = 0.20) ( Figure 2). Haplotype analysis with these two SNP revealed that the C-A haplotype was statistically significantly more prevalent among individuals in the control group compared with centenarian probands (0.355 versus 0.278, *p* <0.004). LD analysis revealed LD across the neighboring apolipoprotein A4 *(APOA4)* and apolipoprotein C3 *(APOC3)* genes as previously reported by others ( Figure 2) [ 8]. In haplotype analysis, the prevalence of the A-C haplotype (corresponding to SNP rs675 in *APOA4* and rs2542052 in *APOC3*) was significantly higher among the centenarian probands compared with controls (0.498 versus 0.344, *p* < 10 ^−8^). The prevalence of the A-A haplotype of the same SNPs was significantly higher among the control group compared with probands (0.483 versus 0.307, *p* < 10 ^−10^). Association analysis between various haplotypes of the *APOC3*/ *APOA4* and lipid profile was not significant, neither when the most prevalent haplotypes in the two groups were compared, (CA versus AA) nor the haplotypes that contain the favorable *APOC3* allele. The only trend for association ( *p* < 0.07) between haplotype and lipoprotein was for LDL. This suggests that the single genotype *APOC3 CC* is more prominent in the phenotypes.

*APOC3* and *CETP* encode gene products involved in lipoprotein metabolism and thus the favorable alleles showing monotonic increases in allele frequencies among older age strata would also be expected to be associated with a favorable lipoprotein profile. Indeed, participants of all groups carrying the *APOC3* −641 CC genotype had lower serum levels of APOC3 compared with those carrying either CA or AA (CA/AA) genotype (mean [SE]: 10.1 [1.1] versus 13.2 [1.1] mg/dl, *p* < 0.05). Given the higher prevalence of this genotype in centenarians, it is therefore not surprising that serum levels of APOC3 were lower in centenarians and their offspring than in controls (mean [SE]: 9.8 [0.6], 9.3 [0.5] versus 11.7 [0.7] mg/dl; *p* < 0.05 and 0.01, respectively) ( Figure 3B).

Relationships between other lipoprotein traits and *APOC3* −641 CC genotype are shown in Table 1. Due to the dependency of lipoprotein traits on age and their modifiability with medications, we only considered offspring ( *n* = 131) and controls ( *n* = 126) not using lipid lowering drugs in this analysis. In females, triglycerides (TG), high density lipoprotein cholesterol (HDL), and their ratio, as well as low density lipoprotein cholesterol (LDL) lipoprotein particle size were significantly more favorable among those with the CC genotype compared to the CA/AA genotypes. The same trends were observed in most of the associations in men. Since there was no significant interaction between gender and genotypes, we present also the combined group of females and males and their significance. As shown in Table 1 the combined group also exhibited significant associations with lipoproteins according to *APOC3 CC* genotype.

**Table 1:**
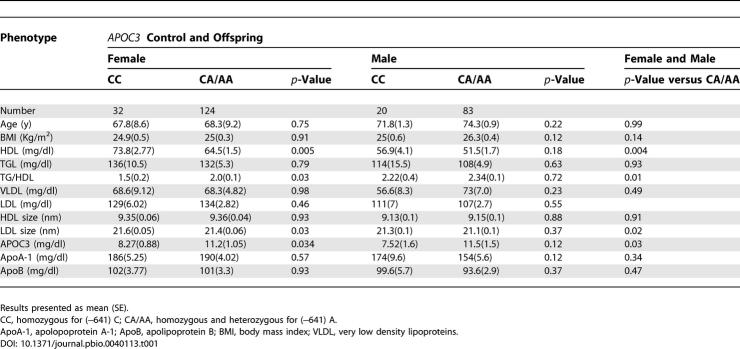
Comparison of Lipoprotein Traits in Females, Males, and All (Combined Offspring and Control Group) Who Were Not on Lipid-Lowering Drugs by *APOC3* C(−641)A Genotype

**Figure 4:**
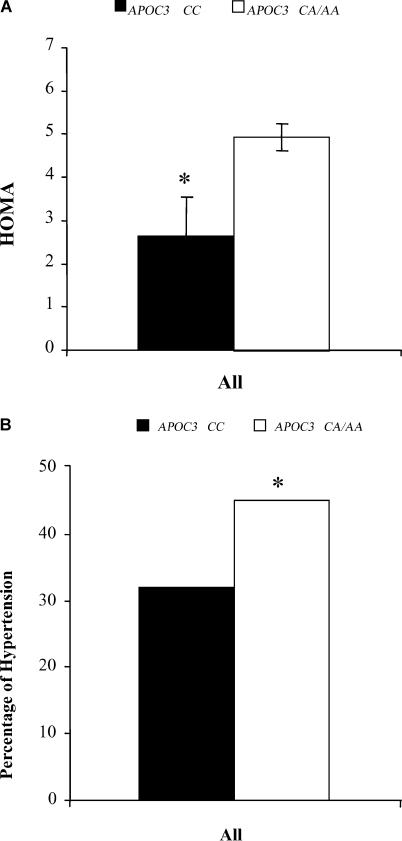
Insulin Sensitivity and Hypertension according to *APOC3* C(−641)A Genotype All include offspring plus controls. (A) Insulin sensitivity. (B) Hypertension. * *p* = 0.05 CC, homozygous for (−641) C; CA/AA, homozygous and heterozygous for (−641) A.

Given that insulin resistance is associated with lipid abnormalities, CVD, and death in the elderly [ 9], we estimated insulin sensitivity using the homeostatic model assessment [ 10]. The favorable *APOC3* −641 CC genotype was associated with greater insulin sensitivity in the combined offspring/control group ( *p* < 0.05, both male and female) ( Figure 4A).

The most prevalent CVD marker in an aging population is progressive increase in blood pressure. Therefore, this trait was examined in our participants as a surrogate for vascular aging. The prevalence of hypertension (as defined in the VII Report of the Joint National Commission on Prevention, Detection, Evaluation, and Treatment of High Blood Pressure) [ 11] was significantly lower among those with the *APOC3* −641 CC genotype than among those with CA/AA genotypes ( *p* = 0.04) ( Figure 4B). Since existing CVD can lead to reductions in blood pressure, we repeated the test after excluding all participants with CVD from the analysis. Still, hypertension was less prevalent in those with the favorable *APOC3 CC* genotype (prevalence of hypertension in participants carrying the *APOC3 CC* genotype was 28.6% compared with 44% in the CA/AA group, *p* = 0.026). Other known variants of the *APOC3* gene: C(−482)T, T(−455)C, C1100T, C3175G, and T3206G, (rs2854117, rs2854116, rs4520, rs5128, and rs4225, respectively) were not associated with TG levels, insulin sensitivity, or blood pressure.

**Figure 5:**
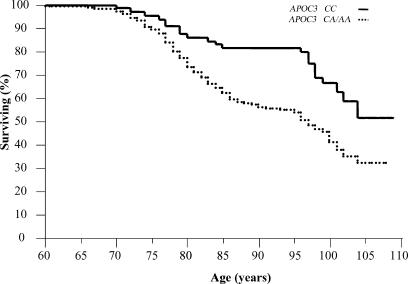
Survival Analysis according to *APOC3* Genotypes Of the 381 participants we genotyped since 1998, 64 had the −641 CC genotype. To describe the relationship between genotype and death, we plotted the Kaplan–Meier survival function estimates of probands and controls by *APOC3* genotype. Offspring were excluded in this analysis because all participants are currently alive and offspring genotype/phenotype is not independent of the probands. Log-Rank ( *p* = 0.0008); Wilcoxon ( *p* = 0.0007). CC, homozygous for (−641) C; CA/AA, homozygous and heterozygous for (−641) A.

Of the 381 participants we have genotyped since 1998, 64 had the CC genotype. To describe the relationship between genotype and death, we plotted the Kaplan-Meier survival function estimates of study participants by *APOC3* genotype ( Figure 5). Survival was significantly greater among CC genotype carriers ( *p* = 0.0007).

## Discussion

APOC3 is a major component of very low density lipoproteins and chylomicron remnants; it is also a minor component of HDL [ 8]. In vitro, it has been shown to inhibit the activity of lipoprotein lipase, resulting in delayed TG clearance from plasma [ 12]. Several studies have shown that various polymorphisms of the *APOC3* gene either within the promoter region, in particular the T-455C variant polymorphism in the insulin response element, or in the gene, are associated with elevated TG levels but not with significant change in APOC3 levels [ 13– 17]. Previous studies have also associated high levels of APOC3 with macrovascular and microvascular disease and their risks. Increased APOC3 levels were a significant indicator of coronary risk in men and in patients with metabolic syndrome. In particular, those with vascular disease and metabolic syndrome had a markedly increased probability of coronary heart disease in an allele-specific way [ 19], and in patients with type 2 diabetes predisposition [ 20]. While few studies have correlated both unfavorable *APOC3* promoter genotypes and high APOC3 levels with outcome [ 21, 22], our study is the first to our knowledge to implicate a genotype with low levels of APOC3 and a favorable lipid phenotype with better outcomes related to longer lifespan. Murine models expressing one to 100 copies of the human *APOC3* gene had high levels of APOC3 and were hypertriglyceridemic [ 23]. *APOC3* knockout mice show absent or decreased APOC3 protein with reduced TG levels [ 24], even in the presence of streptozotocin-induced diabetes [ 25]. The impact on lifespan has not, however, been determined in these models.

We hypothesized that since only one out of 10,000 people in the general population has survived to 100 y, there should be potent enrichment for favorable genotypes in this unique population. The monotonic, progressive over-representation of the *APOC3* −641 CC genotype with increasing age, from a relatively infrequent 10% at age ˜60 y to 25% in individuals surviving to age ˜100 y is statistically robust and provides evidence that this genotype provides a selective advantage for survival to exceptional old age. Underlying population stratification could be responsible for genotypic associations detected in case-control studies, but this is much less likely in this relatively homogeneous Ashkenazi population. Furthermore, we demonstrated association of this same genotype with survival in a prospectively followed cohort. Finally, the prevalence of heterozygosity for recessive disease alleles (nearly) unique to the Ashkenazi population, e.g., breast cancer and Tay-Sachs disease, did not differ significantly between longevity cases and controls, suggesting the lack of population stratification (unpublished data).

In addition to showing statistical associations of the *APOC3* genotype with exceptional longevity, we found significant associations between this genotype and several intermediate longevity-associated traits directly related to lipoproteins. First, serum APOC3 levels were significantly (˜30%) decreased in our participants with the favorable *APOC3* genotype. Second, lipid profiles were altered in the expected (favorable) direction in participants with this genotype. Finally, this favorable genotype was associated with reduced insulin resistance and less hypertension. These findings suggest that the *APOC3* genotype may have favorable pleiotrophic effects on a number of cardiovascular traits and mortality.

Interestingly, while a recent study found that the phenotypic effects of APOC3 polymorphisms are modified by dietary polyunsaturated fatty acids [ 22], the interactions of APOC3 levels with exercise, body mass index or other environmental effect have not been well studied. Our population by genotype had similar body mass index, suggesting that body weight is not strongly modulated by genotype.

The *APOC3* gene is located on the long arm of Chromosome 11q23, in tandem with the apolipoprotein A1 and A4 genes [ 8, 26]. The *APO A1-C3-A4-A5* gene cluster has been studied extensively in relation to lipoproteins and CVD, and most recently in the oldest old [ 8, 27, 28]. Given LD within this gene cluster, we cannot rule out the possibility that the associations we have demonstrated may be due to other functional SNP within this gene cluster. However, our observation that the *APOC3* −641 CC genotype associates with APOC3 levels suggests that this is a functional SNP or is marking a functional SNP within this gene. Further functional studies will be required to determine if this promoter variant directly results in a decrease in transcription.

Our haplotype analysis revealed a 2-SNP haplotype spanning both *APOA4* and *APOC3.* One haplotype, AA in SNPs (rs675 and rs2542052, respectively), was found to be significantly more prevalent among controls, whereas the AC haplotype was significantly more prevalent among centenarians. The association between these two genes and plasma lipids levels in populations has been reported elsewhere [ 8, 27, 28]. Thus, sequence variants in partial LD that constitute one or more haplotypes across more than one of these apolipoprotein genes may influence longevity. However, our findings that Apolipoprotein A1 and Apolipoprotein B levels are not altered by *APOC3* genotype, suggest that the effect of the genotype on the lipoprotein phenotype is mainly mediated by mechanisms that do not involve alterations of these apolipoproteins.

We have also shown an association of the *APOC3* −641 CC genotype with increased insulin sensitivity as measured by homeostatic model assessment. There are known polymorphisms of the *APOC3* promoter within an insulin response element that affect the level of transcription in response to insulin in vitro [ 29]. In vivo, *APOC3* transcription seems to be dependant on insulin, among other factors [ 30]. Although the −641 variant is not located within the insulin response element of the *APOC3* gene, the favorable CC genotype was strongly associated with a better insulin sensitivity estimated by the homeostatic model assessment. This can potentially contribute to healthier aging in individuals with the *APOC3* −641 CC genotype.

While the potential genetic pathways underlying human exceptional longevity remain largely unknown, pathways involved in lipoprotein metabolism appear to have important influences on longevity in humans. First, the CVD-protective phenotypes of high HDL and large lipoprotein particles sizes are dramatically expressed in our centenarians, and were confirmed in Japanese centenarians [ 31], and oldest old Italians [ 32]. Second, a variant in the *CETP* gene [ 6] is over-represented in our centenarians, a finding that was confirmed in oldest old Italians [ 32]. Low CETP levels, which were explained in part by this *CETP* variant, were observed in our study [ 6] and in Japanese centenarians [ 31]. Sequence variants in another lipoprotein gene, the microsomal transfer protein gene, were implicated in another study of centenarians [ 7]. This study adds *APOC3* to the list of lipoprotein genes that may influence exceptional longevity *.* Interestingly, all these genes are involved in different steps of lipoprotein metabolism and could potentially be additive in their effects.

While the concept that alteration of gene expression or function of a particular gene can alter lifespan has been demonstrated convincingly in lower animal models, it is important to determine if those same genes and mechanisms are relevant to the complex biology of mammals and humans. Evidence to support such a connection are the findings that abnormal nematode *daf-16* (a transcription factor) or *daf-2* (insulin/IGF-1 receptor homolog) causes juvenile animals to enter a state of diapause, called dauer, instead of achieving adulthood, thereby extending lifespan [ 33]. Both of these genes are part of the insulin–IGF-I signaling pathway, which has been implicated in longevity in mice [ 34]. Notably the expression of vitellogenin (yolk protein/apolipoprotein-like) genes *(vit-2* and *vit-5)* decreases as lifespan increases [ 35]. This hypothesis has been independently examined in honeybees where the vitellogenin lipoprotein is associated with both modulation of immune function and longevity [ 36]. We suggest that modulation of apolipoprotein expression could represent one conserved mechanism of life extension influencing lifespan across species from nematodes to humans **.** Furthermore, one of the regulators of *APOC3* expression is Forkhead Box O1 *(FOXO-1),* a forkhead transcription factor that mediates insulin action [ 29, 30], which is the homolog of the nematode's *daf-16* [ 33, 37]. Deletion of the *FOXO-1* binding site in mouse hepatocytes annuls the inhibitory action of insulin on hepatic expression of *APOC3,* and animals that constitutively express active *FOXO-1* have hypertriglyceridemia [ 38]. Whether this example is relevant to longevity of mammalian models is not known.

Genetic determinants of human longevity are likely to be multifactorial and polygenic. Thus, polymorphisms in apolipoproteins and related genes are likely to be only one of several potential pathways in which genetic variants influence longevity. Our approach—to exploit the potent selection of favorable genotypes in exceptionally aged individuals—can be extended to other candidate genes as well as genome-wide analysis. Replications of observed associations of genotypes with longevity, coupled with functional studies to define the mechanisms whereby specific genotypes influence longevity-associated phenotypes, will provide important new understandings of killing diseases and the aging process. These new understandings could potentially provide insights into preventive and therapeutic interventions for several age-related diseases that impart significant morbidity and mortality among elderly individuals.

## Materials and Methods

### Study design and participants

In this case-control study, Ashkenazi Jews were recruited as described elsewhere [ 5, 6]. The Ashkenazi population is believed to have descended from a small group of founders based on both historical records and modern genetic evidence. The advantage of using founder populations is that the genetic determinants of a particular disease may be more homogeneous and easier to define, and false positive results due to population stratification is less likely [ 39]. Indeed, specific mutations in a number of disease genes (e.g., *breast cancer gene 1, breast cancer gene 2, adenomatosis polyposis coli gene, hereditary prostrate cancer gene*) have been initially identified in the Ashkenazi population at prevalence rates consistent with a founder effect [ 40– 44]. Stratification in these studies has not been required, and indeed these genes were later implicated in other populations, too [ 45, 46].

Two hundred and thirteen probands with exceptional longevity (157 females and 56 males, age 98.2 (0.36) y [mean (SE)], range 95–107 y; 48% over the age of 100 y) were recruited to participate in the study. The participants' ages were defined by birth certificates or dates of birth as stated on passports. Probands were required to have been living independently when they were 95 y of age as a reflection of good health, although at the time of recruitment they could be at any level of dependency. In addition, probands were required to have a child who was willing to participate in the study. The offspring group consisted of 122 females and 94 males (age 68.3 [0.45] y, range 51–89 y). The control group included 258 participants derived from two sources: Ashkenazi Jews from the general population recruited by the Einstein Aging Study and spouses of the recruited offspring. The study sample included 183 participants with mean age 71.3 (0.67) y, 57% female [ 47]. Spouses of offspring included 75 participants with mean age 70.2 (1.17) y, 53% female. These controls as a comparison group similar in age to the offspring are important to overcome the limitation of a cross-sectional sample, because the effects of age on genotype, phenotype (APOC3 levels, lipids etc.), and outcomes (hypertension and longevity) are minimized. Written informed consent was obtained and the study was approved by the Committee on Clinical Investigations of the Albert Einstein College of Medicine.

### Clinical evaluation

A research nurse visited the research participants in the morning to obtain a medical history, perform a physical examination, and draw a fasting venous blood sample. Standardized health histories were obtained using a questionnaire. At that visit, the offspring and the participating spouses underwent similar evaluations, as previously described [ 5, 6]. Systolic (first phase) and diastolic (fifth phase) blood pressure were obtained twice, using a standard sphygmomanometer with the patient sitting for at least five minutes, and hypertension was defined according to Joint National Commission VII guidelines [ 11]. EAS controls underwent a similar evaluation in an outpatient research center at our institution. All blood samples for centenarians, offspring, and controls were processed at the General Clinical Research Center at Albert Einstein College of Medicine, at the clinical laboratories of Montefiore Medical Center Laboratories (Albert Einstein College of Medicine, Bronx, New York, United States), and at LipoScience (Raleigh, North Carolina, Unites States).

### Lipids and lipoproteins

Total plasma cholesterol, TG, HDL, LDL, APOA1, and apolipoprotein B concentrations for study participants were measured by standard automated methods at the clinical laboratories of Montefiore Medical Center (coefficient of variance [CV] ranged 2.5%–3.5%). APOC3 concentrations in human serum were measured by ELISA using a commercially available kit (Wako Chemicals USA, Richmond, Virginia, United States) (CV ranged 2%–5%).

LDL and HDL subclass levels and mean particle sizes were determined for all participants by proton nuclear magnetic resonance spectroscopy at LipoScience. Each nuclear magnetic resonance measurement produces the concentrations of three LDL subclasses and five HDL subclasses of varying size, as previously described [ 6] (CV ranged between 3.3 and 5.4). Insulin levels were measured by polyclonal immunoassay at the Hormone Assay Core of the Diabetes Research and Training Center of the Albert Einstein College of Medicine (CV of 8.4%).

### *APOC3* genotyping

A multilocus polymerase chain reaction–based assay was utilized to genotype known polymorphisms of *APOC3* on Chromosome 11q: C(−641)A, C(−482)T, T(−455)C, C1100T, C3175G, T3206G. Briefly, DNA was amplified using multiplex reaction containing biotinylated primer pairs. Amplified fragments within each polymerase chain reaction product pool were then detected colorimetrically with sequence-specific oligonucleotide probes immobilized in a linear array on nylon membranes strips. Probe specificities had previously been confirmed by sequencing and by use of DNA genotyped independently through other methods such as restriction length polymorphism analysis [ 48].

### Statistical analyses

Pairwise crude comparisons of lipid levels and lipid particle sizes among the study groups were carried out using the Mann-Whitney U-test because the distributions were skewed. Calculations were carried out using SAS version 6.12 (SAS Institute, Cary, North Carolina, United States) and Stata version 8.2SE (StataCorp, College Station, Texas, United States). Analyses of lipid parameters were stratified by sex, given that there are known differences between female and male lipid profiles and cardiovascular risks [ 49, 50]. Results are expressed as mean (SE).

In order to take into consideration the false positive associations resulting from multiple tests of the 66 SNP ( Table S1), a Bonferroni correction was applied to determine the proper level of statistical significance, *p* = 0.05/66 = 0.0007.

For a comparison of the difference in *APOC3* C(−641)A genotype frequencies between the groups, the chi-square test was performed. Genotype frequencies of all SNP were found to be in Hardy-Weinberg equilibrium. A (corrected) *p*-value less than 0.05 was considered the threshold for statistical significance. Given that this is a relatively homogeneous population, stratification was not applied, except to control for gender effects as noted above. The haplotype association test between cases (probands) and controls was performed using Haploview 3.2 software ( http://www.broad.mit.edu/personal/jcbarret/haploview/index.php) [ 51].

The participants' survival distribution was estimated by the Kaplan-Meier method, and the significance of the difference in survival distribution among the groups was tested by means of a log rank test. Wilcoxon statistics were calculated to test homogeneity between the groups.

## Supporting Information

Table S1 Genes and Their Associated SNP Genotyped for This Study The reported values are percent in Control/Proband and its significance of the change ( *p*-Value).
After Bonferroni correction only *CETP-VV* and *APOC-3 CC* remain significant ( *p* < 0.0066).
(135 KB DOC)

## Abbreviations

APOA-1: apolipoprotein A-1
*APOA4*: apolipoprotein A4 gene
APOC3: apolipoprotein C3
*APOC3*: apolipoprotein C3 gene
*CETP*: cholesterol ester transfer protein gene
CV: coefficient of variance
CVD: cardiovascular disease
DM: diabetes mellitus
HDL: high density lipoprotein cholesterol
LD: linkage disequalibrium
LDL: low density lipoprotein cholesterol
SNP: single nucleotide polymorphisms
TG: triglycerides
VV: valine-valine
