# Supplementary material for: Lipoprotein Genotype and Conserved Pathway for Exceptional Longevity in Humans
Source: PLoS Biol. 2006 Apr 4;4(4):e113. doi: 10.1371/journal.pbio.0040113 (PMC1413567; doi:10.1371/journal.pbio.0040113)
Supplement: Supplementary file 1 [file pbio.0040113.st001.doc]

**Supplementary Information**

**Method and List of genes for which SNPs analysis was performed.**

A multilocus polymerase chain reaction (PCR)-based assay was utilized to genotype known polymorphisms. Briefly, DNA was amplified using multiplex reaction containing biotinylated primer pairs. Amplified fragments within each PCR product pool were then detected colorimetrically with sequence-specific oligonucleotide probes immobilized in a linear array on nylon membranes stripes. Probe specificities had previously been confirmed by sequencing and by use of DNA genotyped independently through other methods such as restriction length polymorphism analysis.

| **Table 1S:** |  |  |  |  |
| --- | --- | --- | --- | --- |
| **Gene symbol** | **Gene name** | **Polymorphism** | **Location** | **Frequency^*^** |
| ACE (DCP1) | angiotensin I-converting enzyme | intron 16 ins/del | 17q23 | 58/43 (0.01) |
| ADD1 | adducin 1; alpha adducin | gly460trp (G/T) | 4p16.3 | 75/74 (0.768) |
| ADRB2 | beta-2-adrenergic receptor | arg16gly (A/G) | 5q32 | 16/21 (0.188) |
|  |  | gln27glu (C/G) | 5q32 | 31/40 (0.04) |
| ADRB3 | beta-3-adrenergic receptor | trp64arg (T/C) | 8p11 | 97/92 (0.01) |
| AGT | angiotensin I | met235thr (T/C) | 1q42 | 41/33 (0.057) |
| AGTR1 | angiotensin II receptor, type 1 | 1166 A/C | 3q24 | 45/48 (0.56) |
| APOA4 | apolipoprotein A-IV | thr347ser (A/T) | 11q23.3 | 70/69 (0.81) |
|  |  | gln360his (G/T) | 11q23.3 | 1/0.5 (0.18) |
| APOB | apolipoprotein B | thr71ile (C/T) | 2p24 | 80/71 (0.03) |
|  |  | arg3500gln (G/A) | 2p24 | 97/100 (0.01) |
| APOC3 | apolipoprotein C-III | -641 C/A | 11q23.3 | 10/25 (0.0001) ^**^ |
|  |  | -482 C/T | 11q23.3 | 61/54 (0.08) |
|  |  | -455 T/C | 11q23.3 | 28/33 (0.18) |
|  |  | 1100 C/T | 11q23.3 | 63/51 (0.007) |
|  |  | 3175 C/G | 11q23.3 | 80/77 (0.35) |
|  |  | 3206 T/G | 11q23.3 | 37/38 (0.84) |
| APOE | apolipoprotein E | cys112arg (T/C) | 19q13 | 82/88 (0.035) |
|  |  | arg158cys (C/T) | 19q13 | 91/84 (0.02) |
| CBS | cystathionine beta-synthase | ile278thr, 68-bp ins | 21q22.3 | 100/100 (1) |
| CETP | cholesteryl ester transfer protein | -631 C/A | 16q21 | 47/34 (0.002) |
|  |  | -629 C/A | 16q21 | 100/87 (0.01) |
|  |  | ile405val (A/G) | 16q21 | 8/23 (0.0001) ^**^ |
|  |  | asp442gly (A/G) | 16q21 | 98/100 (0.12) |
|  |  | intron 14 G(+1)A | 16q21 | 99/100 (0.13) |
|  |  | intron 14 (+3)Tins | 16q21 | 100/100 (1) |
|  |  | intron 1 TaqIB +/- (G/A) | 16q21 | 38/40 (0.7) |
| F2 | coagulation factor II or prothrombin | 20210 G/A | 11p11.1 | 91/94 (0.246) |
| F5 | coagulation factor V | arg506gln (G/A) | 1q23 | 94/95 (0.775) |
| F7 | coagulation factor VII | -323 10-bp del/ins | 13q34 | 3/5 (0.24) |
|  |  | arg353gln (G/A) | 13q34 | 71/67 (0.489) |
| FGB | fibrinogen, beta polypeptide chain | -455 G/A | 4q28 | 59/53 (0.183) |
| GNB3 | guanine nucleotide-binding protein, beta-3 | 825 C/T | 12p13 | 39/50 (0.01) |
| ICAM1 | intercellular adhesion molecule 1 | gly214arg (G/A) | 19p13 | 96/96 (0.658) |
| ITGA2 | integrin, alpha-2 | 873 G/A | 5q11.2 | 39/40 (0.728) |
| ITGB3 | integrin, beta-3 | leu33pro (T/C) | 17q21 | 70/71 (0.73) |
| LDLR | low density lipoprotein receptor | NcoI+/- (A/G) | 19p13.2 | 50/56 (0.19) |
| LIPC | lipase, hepatic | -480 C/T | 15q21 | 3/4 (0.66) |
| LPA | lipoprotein, Lp(a) | 93 C/T | 6q26 | 0.8/1 (0.82) |
|  |  | 121 G/A | 6q26 | 2/4 (0.33) |
| LPL | lipoprotein lipase | -93 T/G | 8p22 | 95/95 (0.51) |
|  |  | asp9asn (G/A) | 8p22 | 97/98 (0.27) |
|  |  | asn291ser (A/G) | 8p22 | 96/97 (0.42) |
|  |  | ser447term (C/G) | 8p22 | 0.6/0.4 (0.75) |
| LTA | lymphotoxin alpha | thr26asn (C/A) | 6p21.3 | 66/64 (0.54) |
| MMP3 | matrix metalloproteinase 3 | -1171 A5/A6 | 11q22 | 45/44 (0.908) |
| MTHFR | 5,10-methylenetetrahydrofolate reductase | 677 C/T | 1p36 | 45/30 (0.0009) |
| NOS3 | nitric oxide synthase 3 (endothelial) | -922 A/G | 7q36 | 62/44 (0.00095) |
|  |  | -690 C/T | 7q36 | 71/86 (0.002) |
|  |  | glu298asp (G/T) | 7q36 | 65/71 (0.214) |
| NPPA | natriuretic peptide precursor A | 664 G/A | 1p36.2 | 94/94 (0.851) |
|  |  | 2238 T/C | 1p36.2 | 80/75 (0.237) |
| PAI1 (SERPINE1) | plasminogen activator inhibitor 1 | -675 G5/G4 | 7q22 | 25/27 (0.613) |
|  |  | 11053 G/T | 7q22 | 30/31 (0.924) |
| PON1 | paraoxonase 1 | met55leu (A/T) | 7q21 | 17/23 (0.17) |
|  |  | gln192arg (A/G) | 7q21 | 37/41 (0.4) |
| PON2 | paraoxonase 2 | ser311cys (C/G) | 7q21 | 2/3 (0.61) |
| PPARG | peroxisome proliferator-activated receptor-γ | pro12ala (C/G) | 3p25 | 93/88 (0.1) |
| SCNN1A | sodium channel, nonvoltage-gated 1 alpha | trp493arg (T/C) | 12p13 | 81/94 (0.0012) |
|  |  | ala663thr (G/A) | 12p13 | 76/63 (0.0015) |
| SELE | selectin E | ser128arg (A/C) | 1q23 | 72/74 (0.779) |
|  |  | leu554phe (C/T) | 1q23 | 84/88 (0.266) |
| TNF | tumor necrosis factor | -376 G/A | 6p21.3 | 95/97 (0.181) |
|  | (TNF superfamily, member 2) | -308 G/A | 6p21.3 | 88/85 (0.335) |
|  |  | -244 G/A | 6p21.3 | 91/83 (0.163) |
|  |  | -238 G/A | 6p21.3 | 92/86 (0.195) |

Table 1S: Genes and their associated SNPs genotyped for this study.

^*^ The reported values are percent in Control/Proband and its significance of the change (*P*-Value)

^**^ After Bonferroni correction Only *CETP-VV* and *APOC-3 CC* remain significant (*P*<0.0066).
